# Round the Hospitals

**Published:** 1921-03-05

**Authors:** 


### ROUND THE HOSPITALS.

The retirement of Miss F. E. Nevile from her post as matron of the West London Hospital is the subject of widespread regret among her nursing sisters and all who have been associated with her for so long in conducting the affairs of the hospital. She has been twenty years in the institution, for sixteen of which she was matron, and during her time of office the training-school has moved forward steadily and gained a high reputation. Perhaps her greatest successes were won in the troubled period during which she had to find rough-and-ready accommodation for her nurses in several different houses remarkably ill-adapted for the purposes of a nurses' home. It was a triumph of no mean order to maintain discipline and keep a high tone prevailing among probationers under such conditions, but her staff, following her lead, rose to the spirit of the occasion, made light of discomforts, and turned all their difficulties to good account. Miss Nevile's unfailing determination and power of kindling enthusiasm in others prevailed in due course, and she leaves the nursing staff in possession of quarters which are creditable to the hospital. On February 22 a numerous company, including Lord and Lady Arthur Hill, Lady Plender, the Mayor of Hammersmith, and other influential supporters of the West London Hospital, gathered in the Board Room to take a formal farewell of the matron, and Mr. G. F. Marshall, Chairman of the Board, presented Miss Nevile with two cheques, one from a number of friends and another from the hospital. The new matron, as we announced in our issue of December 4, is Miss M. K. Coggins, formerly matron at the Wigan Royal Albert Edward Infirmary.

The inauguration of the eight-hour day at Addenbrooke's Hospital, Cambridge, marks an epoch in the development of this famous training-school. Formerly it could choose among a multitude of good candidates, but there has been a sad falling-off here, as elsewhere, and improved conditions of service were unmistakably indicated. At the recent quarterly Court held at the hospital the Committee announced that the eight-hour day has been adopted for the nursing staff, that the salaries have been raised, and a more liberal allowance for uniform granted. Eight additional probationers have been engaged, and for their accommodation No. 3 Addenbrooke's Place, a house which belongs to the hospital, has been furnished as an annexe to the nurses' home. Dr. Pearce, in commenting on the announcement, declared that if anybody deserved to have fixed hours it was the nurses, who were so often called on to do too much.

Considerable interest has been excited by the libel action by Miss E. H. Grime and Miss A. Rainforth, matron and sister of the Hornsey Cottage Hospital, against Dr. Peter Ingram. On May 28, 1919, a patient called Archell in this hospital underwent an operation on the knee-joint at the hands of Dr Ingram. The patient died and the defendant made certain allegations as to the nursing into which the house committee of the hospital inquired. The matron was on holiday duty at the time of the operation, but returned shortly before the patient died. The house committee exonerated both her and Miss Rainforth, who was acting matron in her absence, from the charge of neglect; but Dr. Ingram wrote on July 1 to Mr. J. Scott Balfour, Chairman of the Hospital, pressing

**ROUND THE HOSPITALS—(continued).**

serious complaints as to the nursing of the case, and also wrote to the *Hornsey Journal* to the same effect. Miss Grime contended that the letters suggested that she was careless and inefficient in her supervision of the case, and was not fitted to be matron of the hospital. A good deal turned on difficulties connected with keeping the bed dry under the Carrel-Dakin treatment and the advisability of offering a patient roast mutton and potatoes on the day following the operation. In the end the jury, on which three women were sitting, disagreed. We are informed that Dr. Ingram was defended by the London and Counties Medical Protection Society.

---

The Southwark Guardians are to be congratulated on their attitude towards their hospital committee. The chairman of this committee, which is responsible for the administration of the Southwark Infirmary, is Miss Gow, and at the last meeting of the Guardians it was her duty to report that three probationers have asked to be relieved from their terms of engagement, to which assent has been given. Others of the staff who gave notice to terminate engagements are two probationers, the night superintendent, and two staff nurses. The reason for these resignations was not explained by Miss Gow in her report, which was accepted by the Board without comment. This, we contend, was the proper course to pursue, and an example of the right method of governing public institutions.

---

Sir Nevil Macready is in favour of increasing the staff of the women police and entrusting to them the entire control of that difficult class in the community euphemistically termed "unfortunates." The first gleams of something approaching, we will not say success in this direction, but lessened failure, were observable in the labours of the women's patrol officers during the war, and following up this line of advance some more hopeful outlook may possibly be anticipated. Sir Nevil seems to be confident that the right kind of woman can be attracted to this work, and he believes nurses would be admirably suited for it.

---

A good deal of friction has arisen in the county of Hampshire owing to the overlapping of health visitors' duties with those of the district nurses. As usual, it is the organisations rather than the individuals which disagree. The Hampshire County Nursing Association considers its nurses are slighted by being put under health visitors frequently less well qualified than themselves. The Health and Housing Committee hold strongly to their health visitors, of whom they have thirty for tuberculosis, school-work, and V.D. The incalculable waste of time involved in sending health visitors to visit tuberculosis cases and school children in districts where a nurse is in residence does not seem to have met with consideration. Our sympathies are decidedly with the district nurses. Some co-ordination of the rival systems is urgently called for.

The demand for health visitors continues. The Health Committee of the Durham County Council has been considering the question of the appointment of additional visitors, but finds difficulty in securing suitable candidates owing to the higher salaries being paid elsewhere. In the circumstances the Committee proposes to fix the salaries of health visitors at such an amount as, with the bonus, will raise their minimum salary to £260 per annum, rising by annual increments of £10 to a maximum of £300, including uniform and subsistence allowances. The Committee also recommends that the Superintendent Health Visitor be granted an increase of salary which, with bonus, will raise her minimum to £400 a year, rising to a maximum of £450. In the case of partially-trained health visitors, it is suggested that their salary should be limited to £260 per annum, and that they shall be informed that unless they qualify within a period of two years the question will be considered of terminating their appointments. We commend these salaries to the notice of the Dudley Corporation Maternity and Child Welfare Committee.

---

Some curiosity has been excited about the number of the *League News* of St. Bartholomew's nurses which was buried by the Queen under the foundation-stone of the new Nurses' Home. It appears to have been the issue of September last, which contains, together with a list of the League members and other information, the complete text of the Nurses' Registration Act, 1919. The *League News* aims at publication twice yearly, though we are informed that in 1920 only one number was issued.

---

It now appears that we were justified in repelling the suggestion circulated in the French papers that Miss Cavell's friend, Madamoiselle Louise Thulliez, had taken her own life as a sequel to denunciation as a German spy. The unhappy lady who has committed suicide is Mlle. Tellier, who was involved in the same trial.

---

The Friends' Relief Mission is doing magnificent work in fighting typhus in Poland, and we learn from an article in the *Manchester Guardian* that in one centre of their work, two thousand feet up in the Carpathian Mountains they have set afoot what is acknowledged to be the only clean hospital in Poland. When the Friends took it over its condition was indescribably filthy; a few most undesirable women and one man formed the staff, and no women of good character would attempt nursing. Now there is an English sister in charge, and it is a model institution. A willing band of Polish girls has gathered round the sister, and they have already learned to obey and to serve. There are hopes that from this beginning something approaching to a training-school may be evolved. It is good to reflect upon the many centres of life and leading being formed all over the world by nursing sisters imbued with the ideals of their own British training-schools.

